# Supplementary material for: Proximal Molecular Probe Transfer (PROMPT), a new approach for identifying sites of protein/nucleic acid interaction in cells by correlated light and electron microscopy
Source: Sci Rep. 2023 Dec 5;13:21462. doi: 10.1038/s41598-023-45413-8 (PMC10697944; doi:10.1038/s41598-023-45413-8)

Supplementary Information

Proximal Molecular Probe Transfer (PROMPT), a new approach for identifying sites of protein/nucleic acid interaction in cells by correlated light and electron microscopy

Guillaume A Castillon[1], Sebastien Phan[1], Junru Hu[1], Daniela Boassa[1], Stephen R Adams[2], Mark H Ellisman* [1, 3,]

[1]Department of Neurosciences, University of California San Diego, La Jolla, CA 92093, USA.

[2]Department of Pharmacology, University of California San Diego, La Jolla, CA 92093, USA.

[3]Center for Research in Biological Systems, National Center for Microscopy and Imaging Research, University of California San Diego, La Jolla, CA 92093, USA.

* Corresponding author, mellisman@health.ucsd.edu

Figure S1: Photosensitizer spectra used in PROMPT.

Figure S2: Synthesis of TMR-PROMPT and TMR-PEG4-PROMPT

Figure S3: Synthesis of JF570-PROMPT and JF525-PROMPT

Figure S4: Enhanced brightness of the images displayed in Figure 3b

Figure S5: Fibrillarin/RNA PROMPT with JF525-PROMPT. Confocal micrographs of U2OS cells transfected with or without the HaloTag-Fibrillarin construct, pretreated overnight with or without 1mM 5EU, and processed with the PROMPT procedure using JF525-PROMPT.

Figure S6: Enlargement of the Cajal body from Figure 7b

LC-MS chromatograms and spectra

Figure S7-S16

Supplementary movie 1: Multi-tilt axis electron tomogram of a U2OS cell expressing HaloTag-H2B pretreated overnight with 5uM of EdU, processed with the PROMPT procedure and DAB photo-oxidized. The total tomogram thickness is 250 nm.

Supplementary movie 2: Multi-tilt axis electron tomogram of a U2OS cell expressing HaloTag-Fibrillarin pretreated overnight with 1uM of 5EU, processed with the PROMPT procedure and DAB photo-oxidized. The total tomogram thickness is 250 nm.

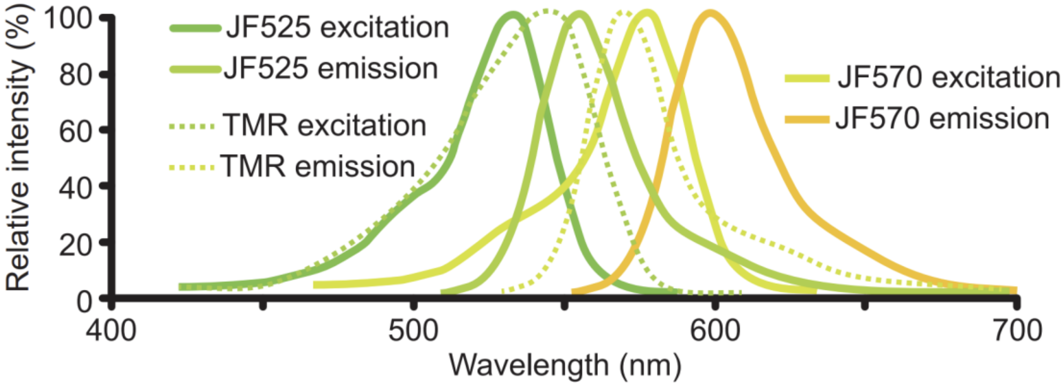

Figure S1: Photosensitizer spectra used in PROMPT.

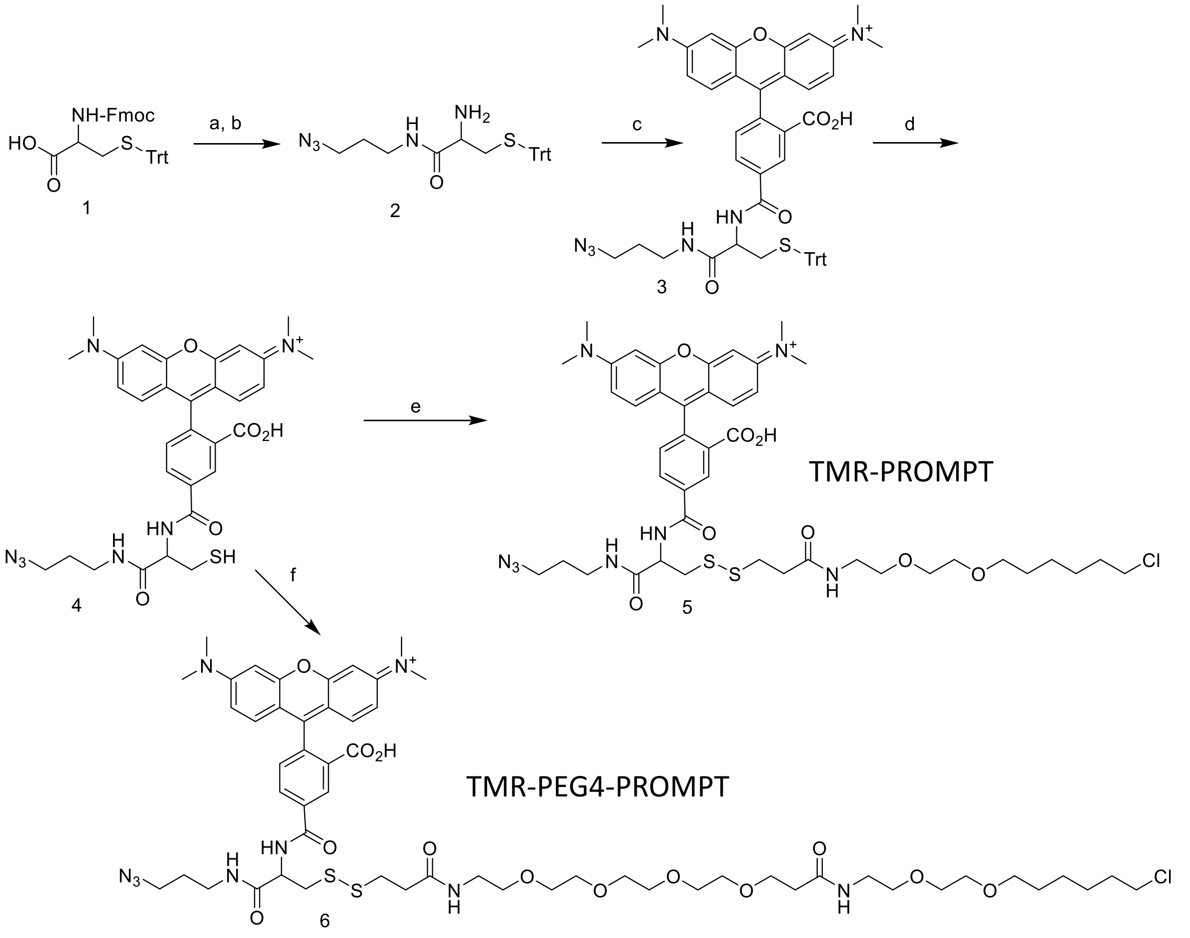

a = 3-azidopropyl-1-amine, HATU, DIEA, DMF; b, piperidine; c, 5,6-carboxy-tetramethylrhodamine, TSTU, DIEA; d, TFA, TIPS, EDT, H_2O; e, 2-Pyridyl-SS-$(CH_2)_2$CONH$(CH_2)_2$O$(CH_2)_2$O$(CH_2)_6$Cl; f, 2-Pyridyl-SS-$(CH_2)_2$CONH-peg$_4$-CONH$(CH_2)_2$O$(CH_2)_2$O$(CH_2)_6$Cl

Synthesis of TMR-PROMPT and TMR-PEG4-PROMPT. Carboxytetramethylrhodamine is a mixture of 5- and 6-isomers; only one isomer is shown for clarity

Figure S2: Synthesis of TMR-PROMPT and TMR-PEG4-PROMPT

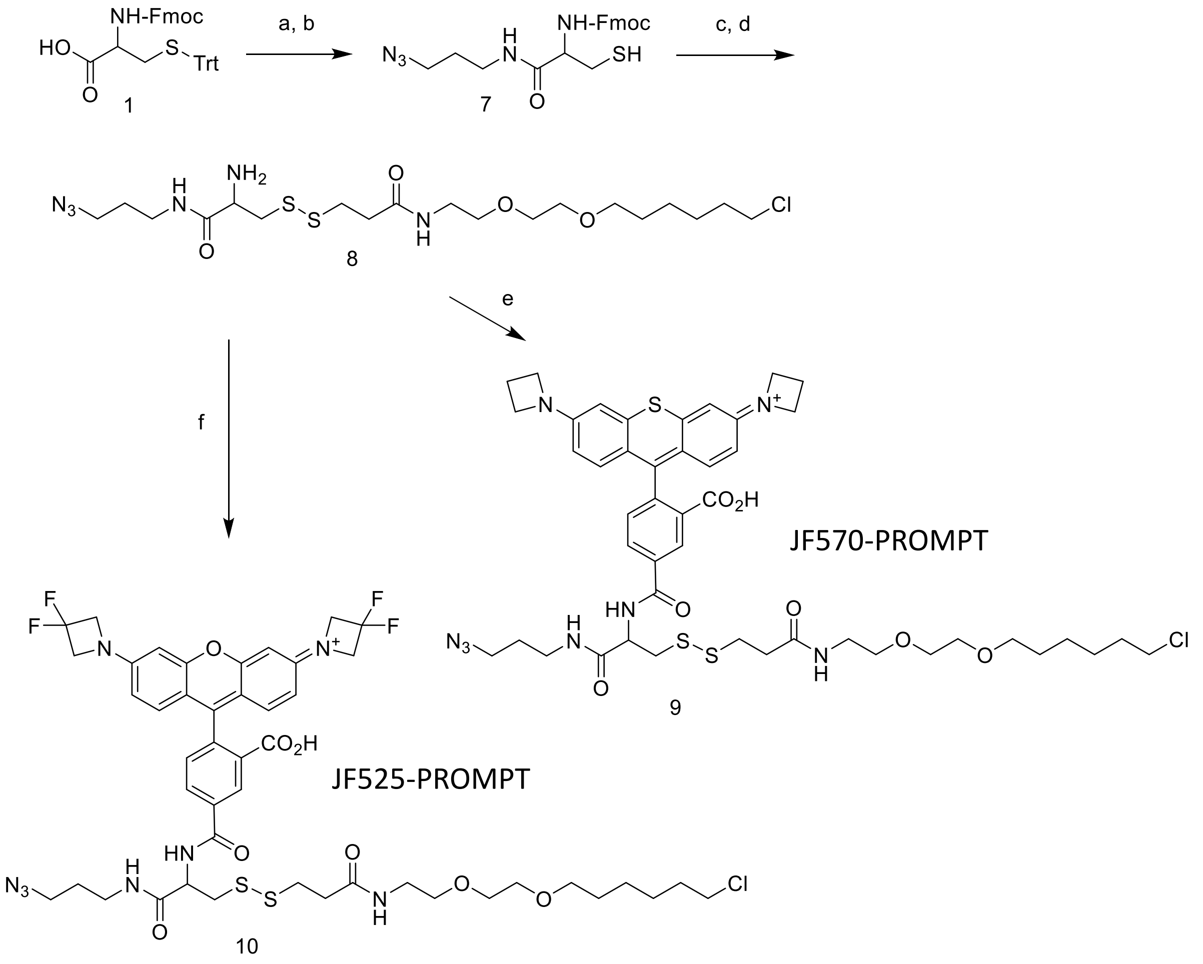

a, 3-azidopropyl-1-amine, HATU, DIEA, DMF; b, TFA, TIPS, EDT, H_2O; c, 2-Pyridyl-SS-$(CH_2)_2CONH(CH_2)_2O(CH_2)_2O(CH_2)_6Cl$; d, piperidine; e, JF525-NHS; f, JF590-NHS

Figure S3: Synthesis of JF570-PROMPT and JF525-PROMPT

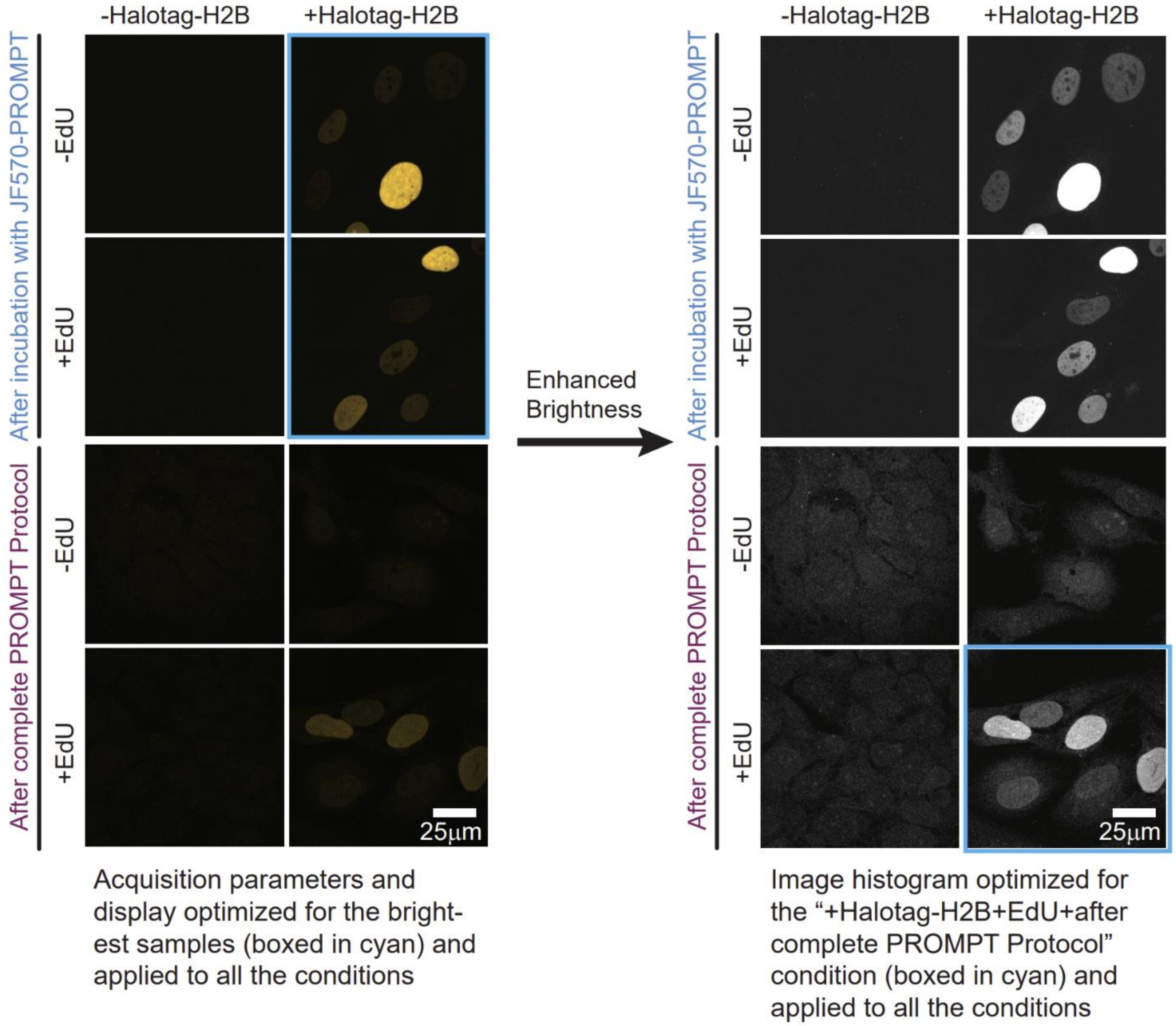

Figure S4: Enhanced brightness of the images displayed in Figure 3b

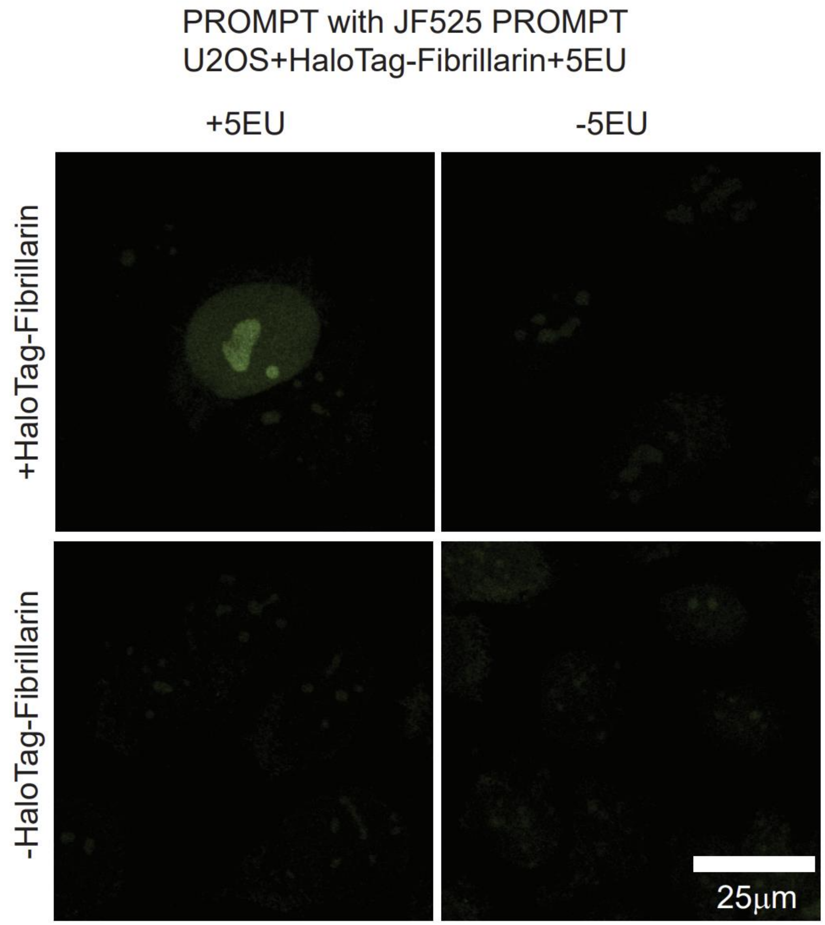

Figure S5: Fibrillarin/RNA PROMPT with JF525-PROMPT. Confocal micrographs of U2OS cells transfected with or without the HaloTag-Fibrillarin construct, pretreated overnight with or without 1mM 5EU, and processed with the PROMPT procedure using JF525-PROMPT.

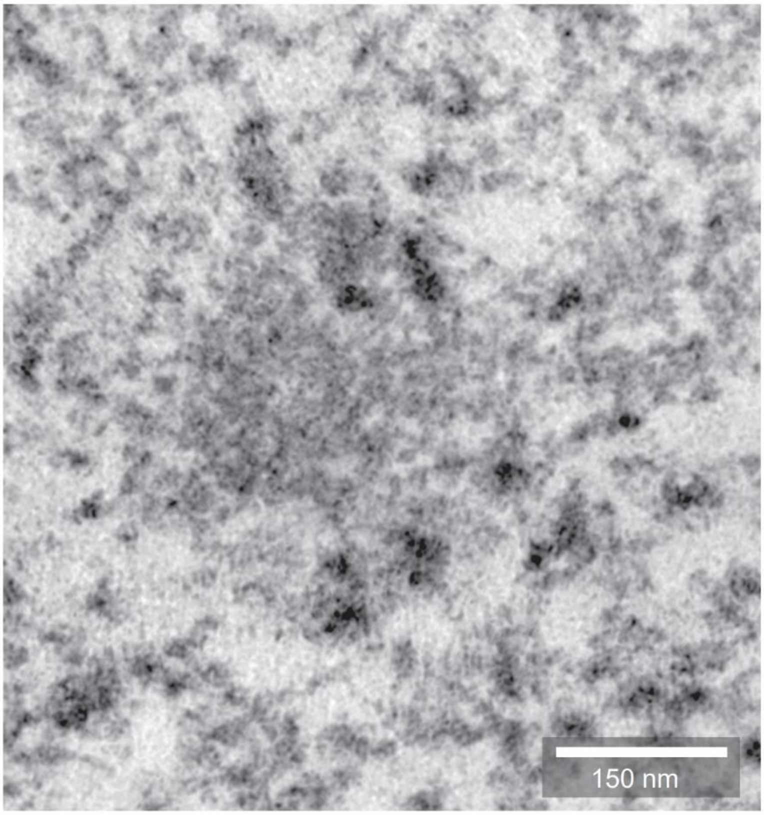

Figure S6: Enlargement of the Cajal body from Figure 7b

LC-MS chromatograms and spectra

Figure S7. Positive ion electrospray liquid chromatography mass spectroscopy analysis of NH_2-cys(S-Trt)-CO-NH-$(CH_2)_3$-N_3 **(2)**. Linear gradient from 10-100% acetonitrile-water-0.05% TFA in 20mins, then held for 5 mins. 1 ml/min.

Figure S8. Positive ion electrospray liquid chromatography mass spectroscopy analysis of 5(6)-TMR-CONH-cys(STrt)-CO-NH-$(CH_2)_3$-N_3 **(3).**
Linear gradient from 5-100% acetonitrile-water-0.05% TFA in 20mins, then held for 5 mins. 1 ml/min

Figure S9. Positive ion electrospray liquid chromatography mass spectroscopy analysis of 5(6)-TMR-CONH-cys(SH)-CO-NH-$(CH_2)_3$-N_3 **(4).**
Linear gradient from 5-100% acetonitrile-water-0.05% TFA in 20mins, then held for 5 mins. 1 ml/min.

Figure S10. Positive ion electrospray liquid chromatography mass spectroscopy analysis of TMR-PROMPT; 5(6)-TMR-CONH-cys(S-S-$(CH_2)_2$CONH-HaloTag ligand)-CO-NH-$(CH_2)_3$-N_3 **(5).** Linear gradient from 5-100% acetonitrile-water-0.05% TFA in 20mins, then held for 5 mins. 1 ml/min.

Figure S11. Positive ion electrospray liquid chromatography mass spectroscopy analysis of TMR-PEG4-PROMPT; 5(6)-TMR-CONH-cys(S-S-$(CH_2)_2$CONH-PEG4-HaloTag ligand)-CO-NH-$(CH_2)_3$-N_3 **(6).** Linear gradient from 5-100% acetonitrile-water-0.05% TFA in 20mins, then held for 5 mins. 1 ml/min.

Figure S12. Positive ion electrospray liquid chromatography mass spectroscopy analysis of Fmoc-NH-cys(SH)-CO-NH-$(CH_2)_3$-N_3 **(7).** Linear gradient from 5-100% acetonitrile-water-0.05% TFA in 20mins, then held for 5 mins. 1 ml/min.

Figure S13. Positive ion electrospray liquid chromatography mass spectroscopy analysis of NH_2-cys(S-S-2-$(CH_2)_2$CONH-HaloTag ligand)-CO-NH-$(CH_2)_3$-N_3 **(8).** Linear gradient from 10-100% acetonitrile-water-0.05% TFA in 20mins, then held for 5 mins. 1 ml/min.

Figure S14. Positive ion electrospray liquid chromatography mass spectroscopy analysis of JF525-PROMPT; Janelia Fluor 525-CONH-cys(S-S-$(CH_2)_2$CONH-HaloTag ligand)-CO-NH-$(CH_2)_3$-N_3 **(10).** Linear gradient from 10-100% acetonitrile-water-0.05% TFA in 20mins, then held for 5 mins. 1 ml/min.

Figure S15. Positive ion electrospray liquid chromatography mass spectroscopy analysis of JF570-PROMPT (**9**) and reaction of JF570-PROMPT with HaloTag protein. Linear gradient from 20-60% acetonitrile-water-0.05% TFA in 16mins, then to 90% in 4 min, and held for 5 mins. 1 ml/min PLRP-S column.

Figure S16. Positive ion electrospray liquid chromatography mass spectroscopy analysis of reaction of JF525-PROMPT with HaloTag protein. Linear gradient from 20-60% acetonitrile-water-0.05% TFA in 16mins, then to 90% in 4 min, and held for 5 mins. 1 ml/min PLRP-S column

Figure S7. Positive ion electrospray liquid chromatography mass spectroscopy analysis of NH_2-cys(S-Trt)-CO-NH-$(CH_2)_3$-N_3 **(2)**. Linear gradient from 10-100% acetonitrile-water-0.05% TFA in 20mins, then held for 5 mins. 1 ml/min

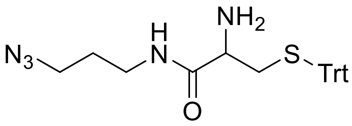

2

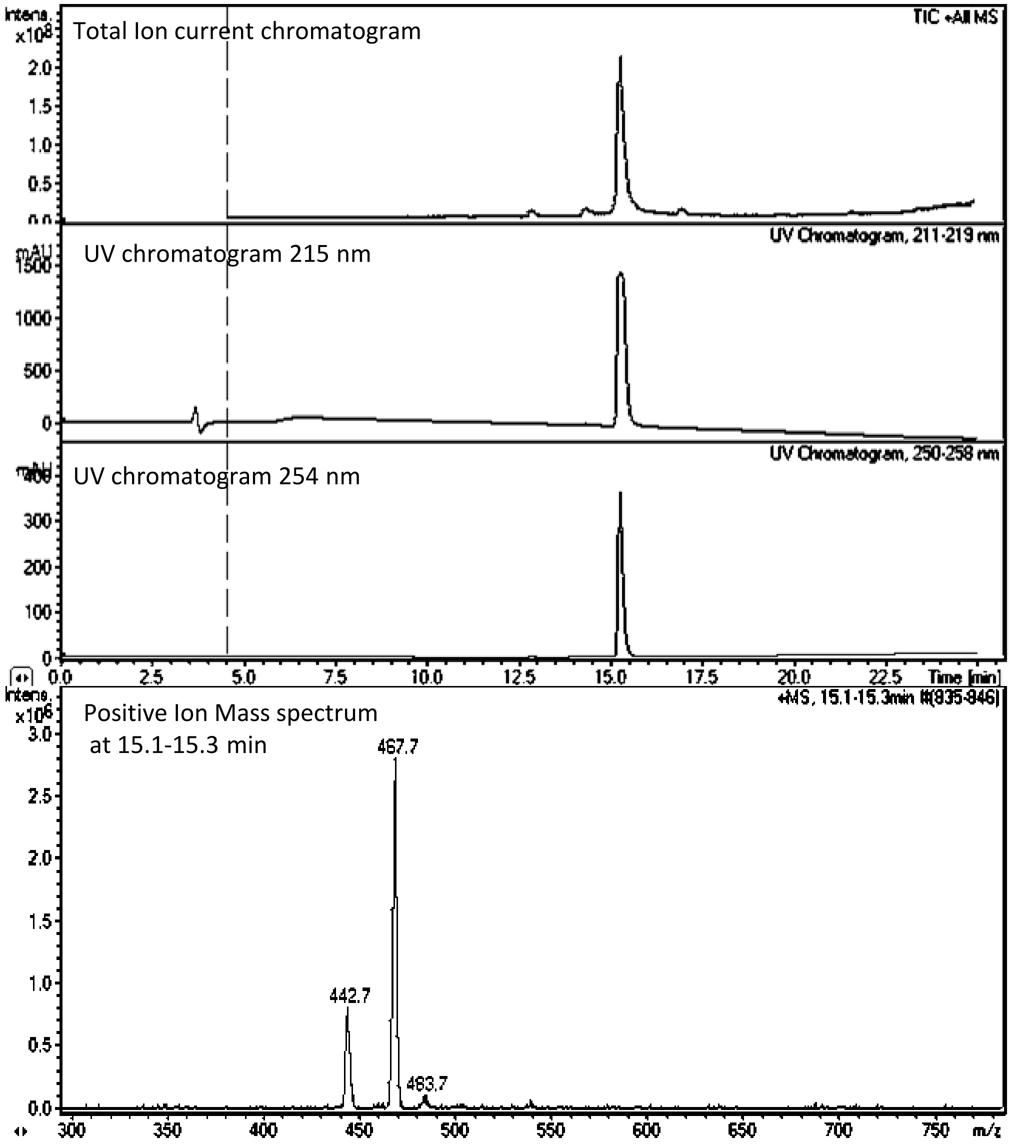

Figure S8. Positive ion electrospray liquid chromatography mass spectroscopy analysis of 5(6)-TMR-CONH-cys(STrt)-CO-NH-$(CH_2)_3$-N_3 **(3).** Linear gradient from 5-100% acetonitrile-water-0.05% TFA in 20mins, then held for 5 mins. 1 ml/min

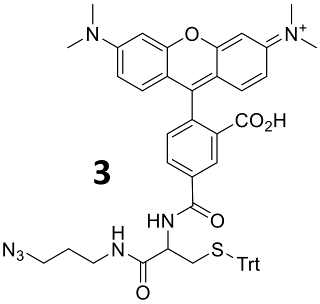

3

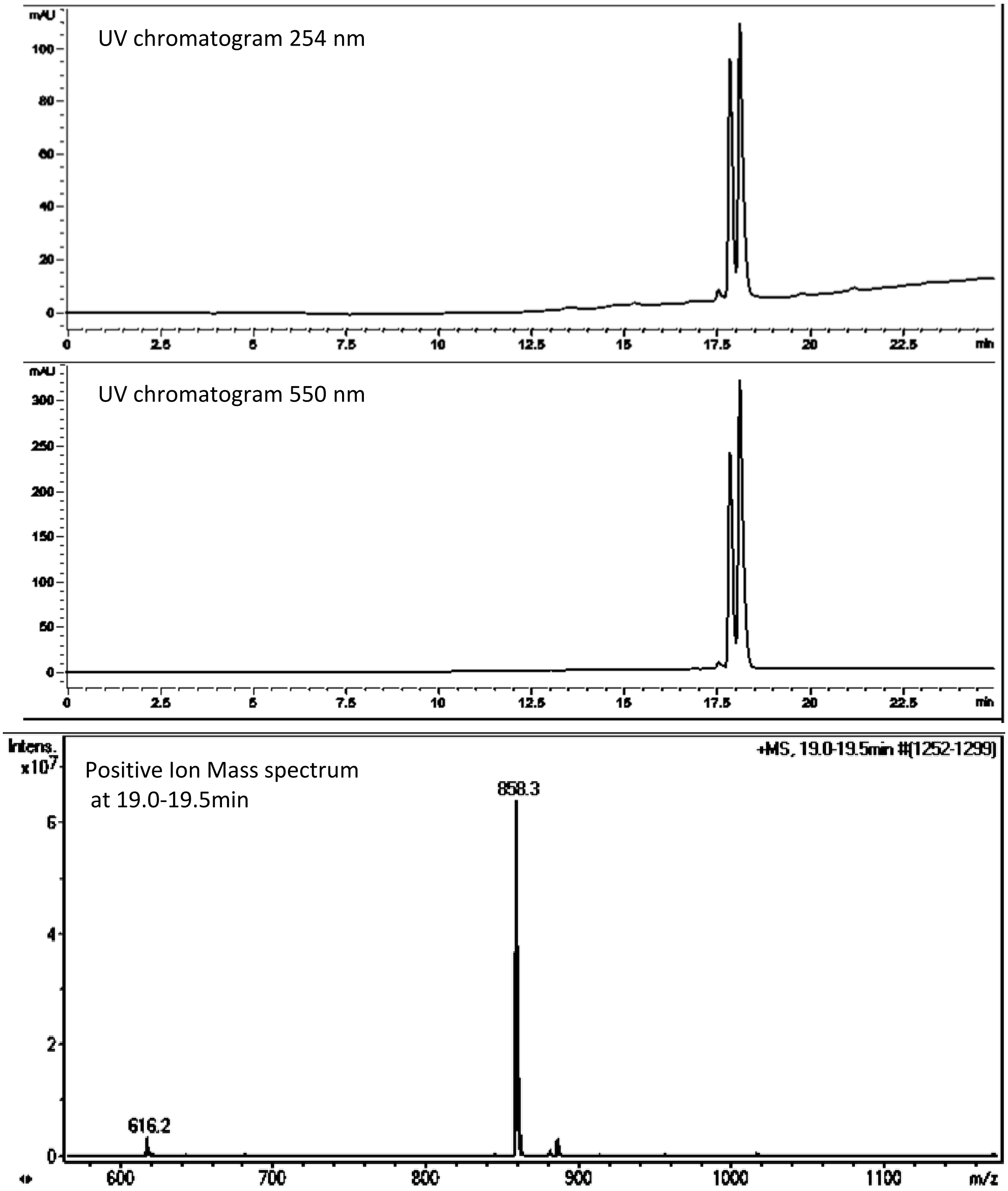

Figure S9. Positive ion electrospray liquid chromatography mass spectroscopy analysis of 5(6)-TMR-CONH-cys(SH)-CO-NH-$(CH_2)_3$-N_3 **(4).** Linear gradient from 5-100% acetonitrile-water-0.05% TFA in 20mins, then held for 5 mins. 1 ml/min

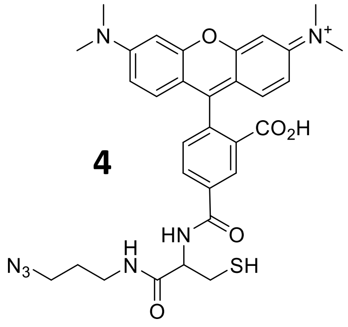

4

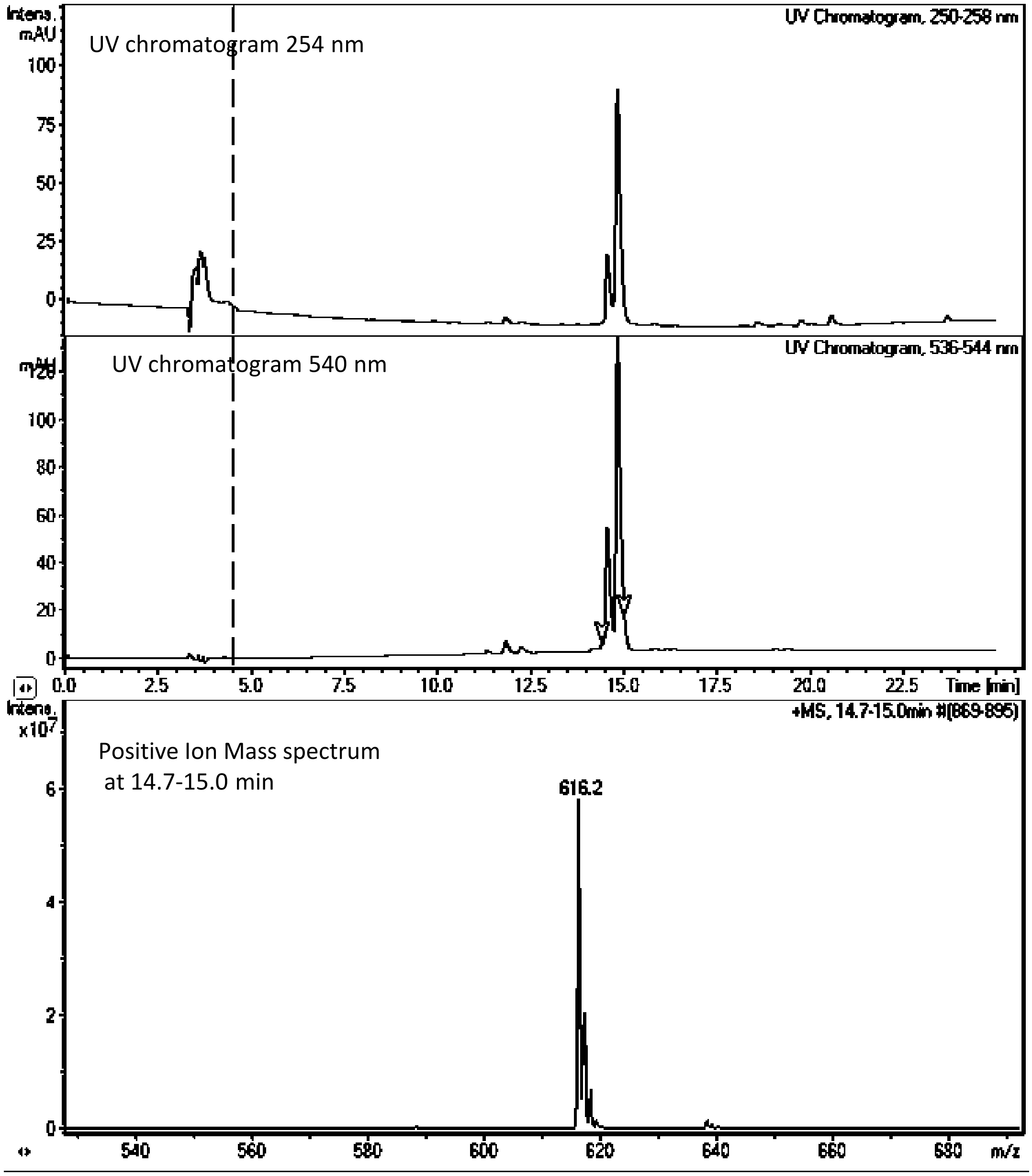

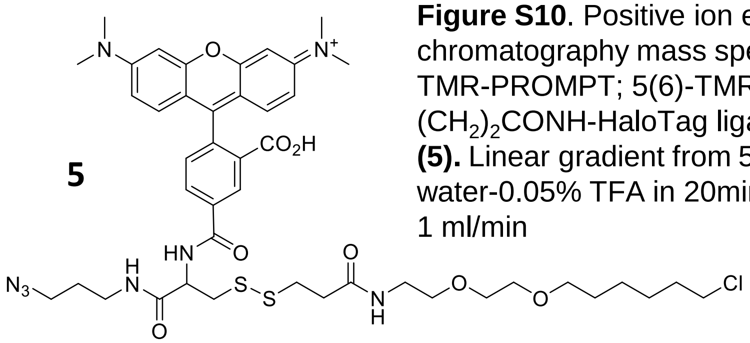

5

Figure S10. Positive ion electrospray liquid chromatography mass spectroscopy analysis of TMR-PROMPT; 5(6)-TMR-CONH-cys(S-S-$(CH_2)_2$CONH-HaloTag ligand)-CO-NH-$(CH_2)_3$-N_3 **(5).** Linear gradient from 5-100% acetonitrile-water-0.05% TFA in 20mins, then held for 5 mins. 1 ml/min

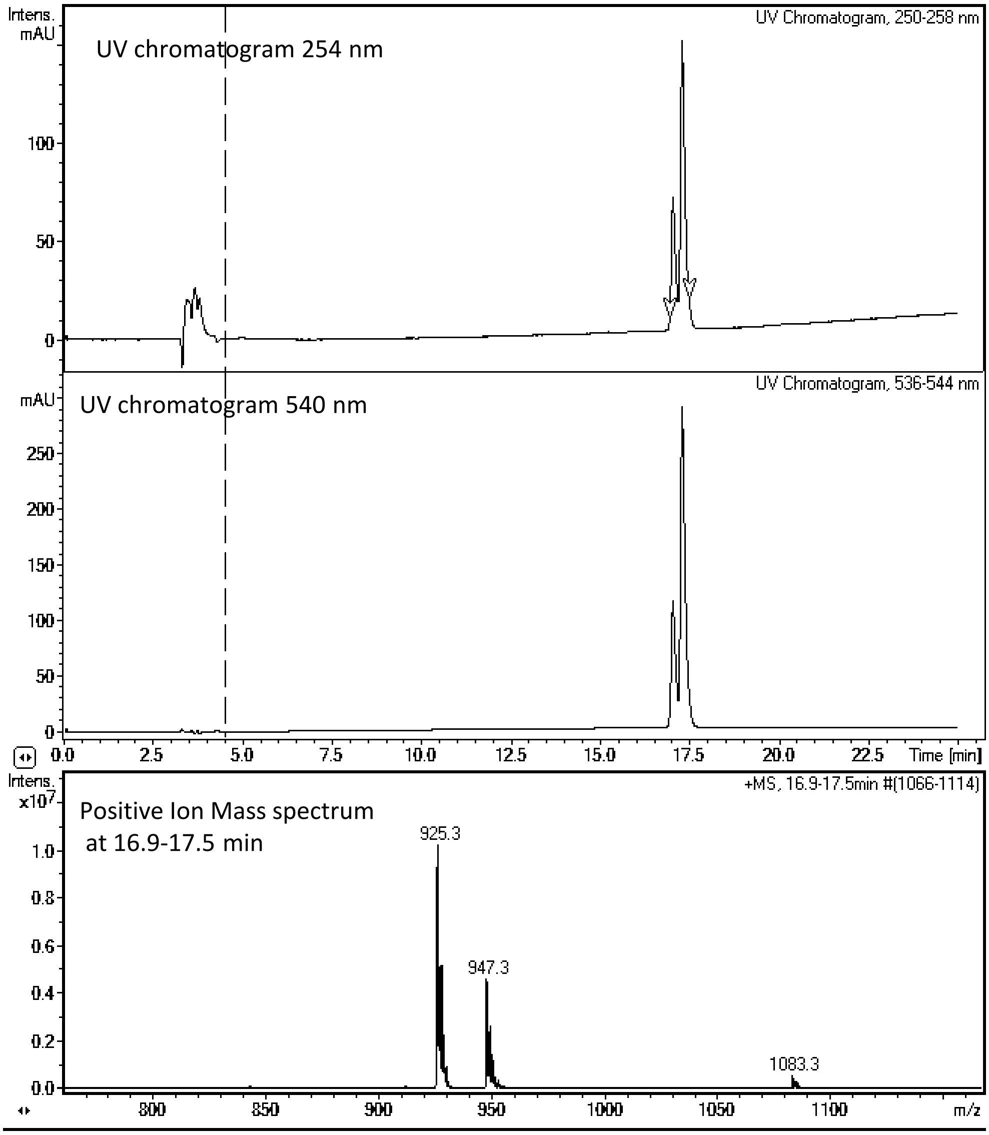

Figure S11. Positive ion electrospray liquid chromatography mass spectroscopy analysis of TMR-PEG4-PROMPT; 5(6)-TMR-CONH-cys(S-S-$(CH_2)_2$CONH-PEG4-HaloTag ligand)-CO-NH-$(CH_2)_3$-N_3 **(6).** Linear gradient from 5-100% acetonitrile-water-0.05% TFA in 20mins, then held for 5 mins. 1 ml/min

6

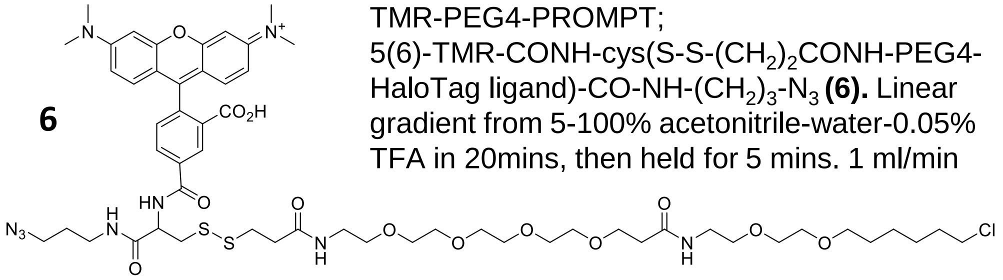

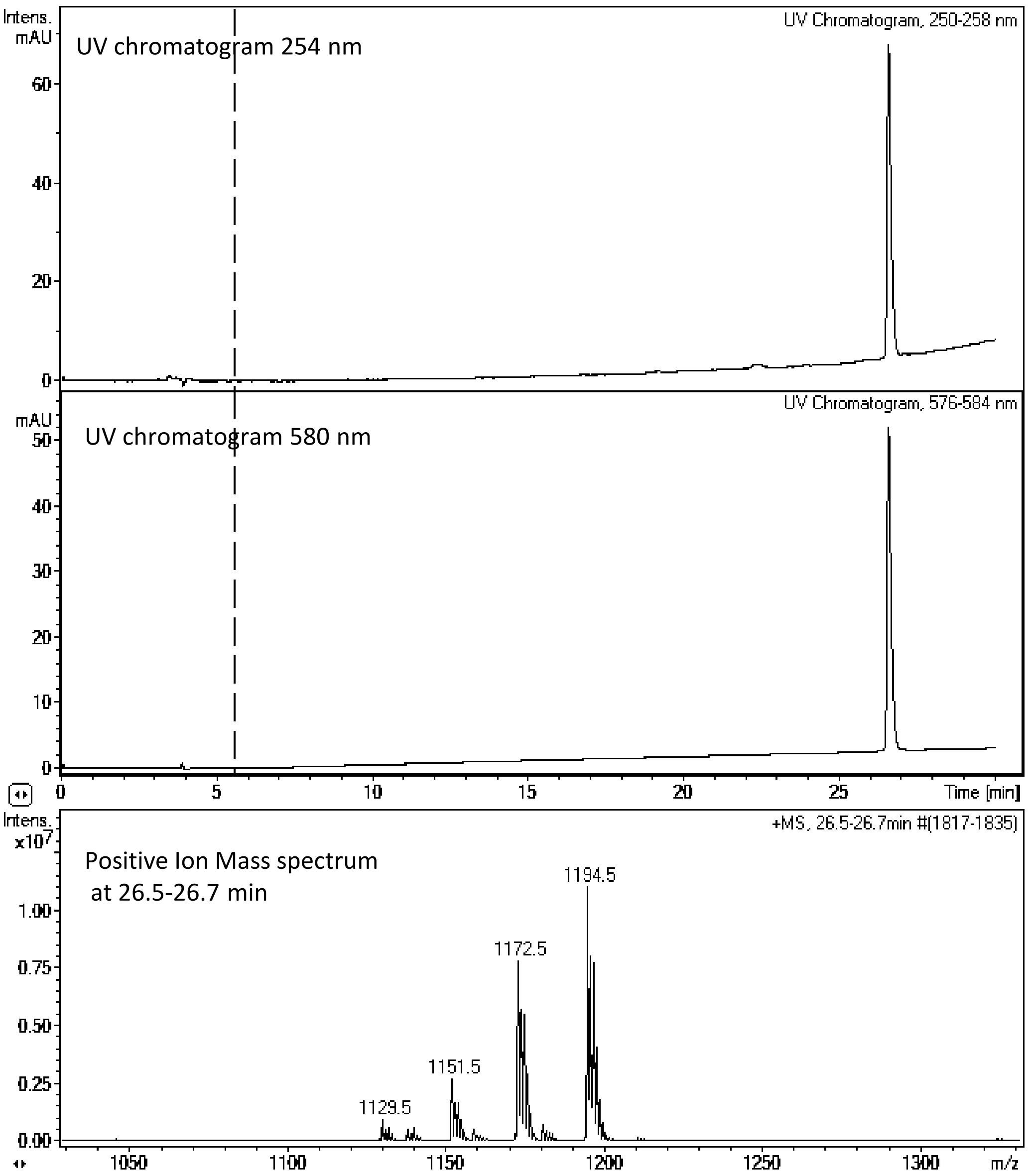

Figure S12. Positive ion electrospray liquid chromatography mass spectroscopy analysis of Fmoc-NH-cys(SH)-CO-NH-$(CH_2)_3$-N_3 **(7).** Linear gradient from 5-100% acetonitrile-water-0.05% TFA in 20mins, then held for 5 mins. 1 ml/min

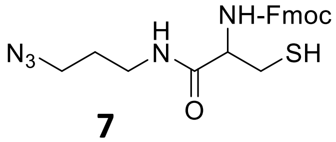

7

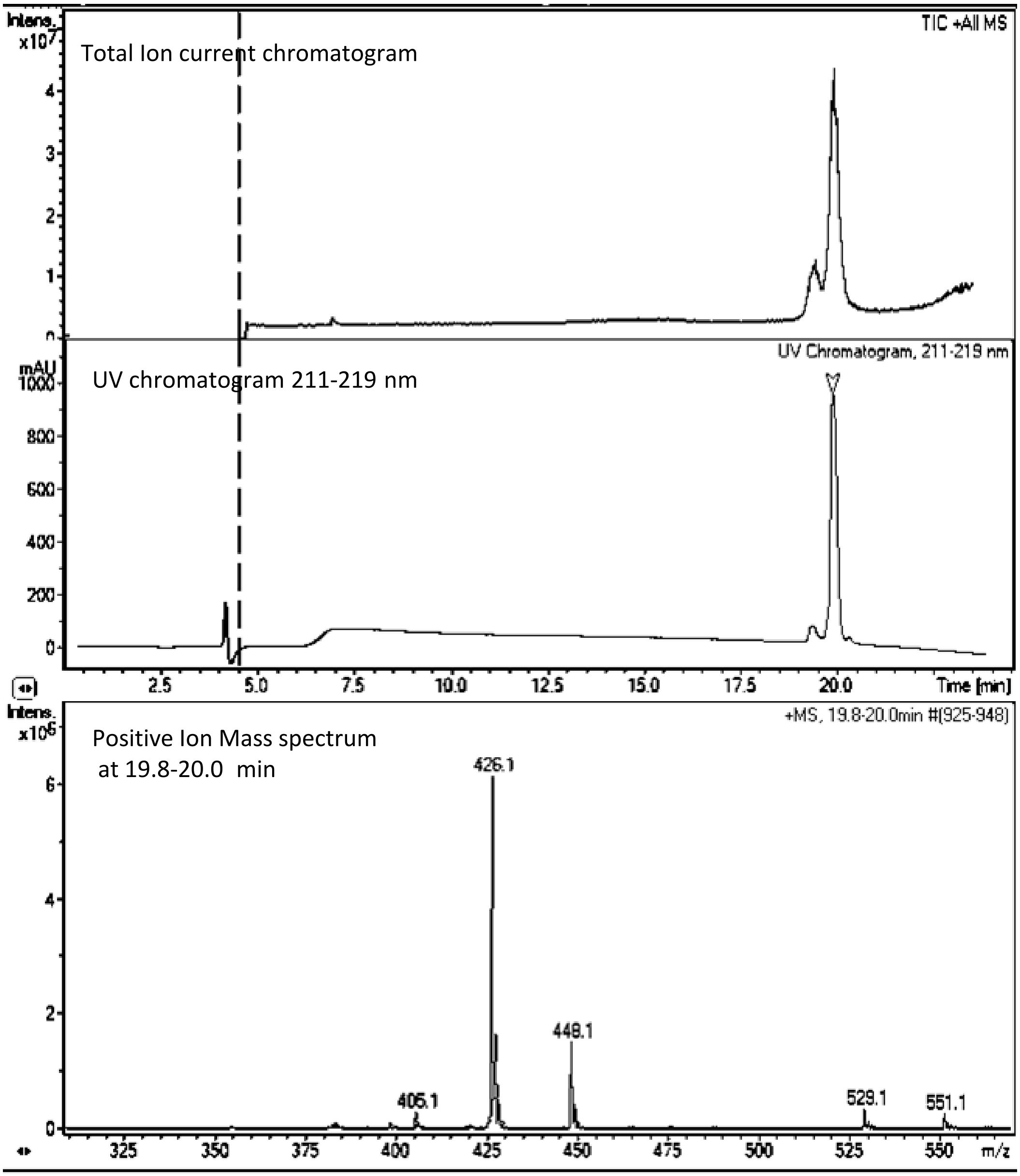

Figure S13. Positive ion electrospray liquid chromatography mass spectroscopy analysis of NH_2-cys(S-S-2-$(CH_2)_2$CONH-HaloTag ligand)-CO-NH-$(CH_2)_3$-N_3 **(8).** Linear gradient from 10-100% acetonitrile-water-0.05% TFA in 20mins, then held for 5 mins. 1 ml/min

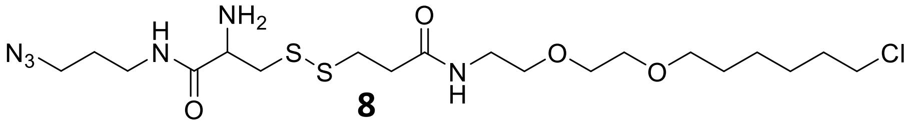

8

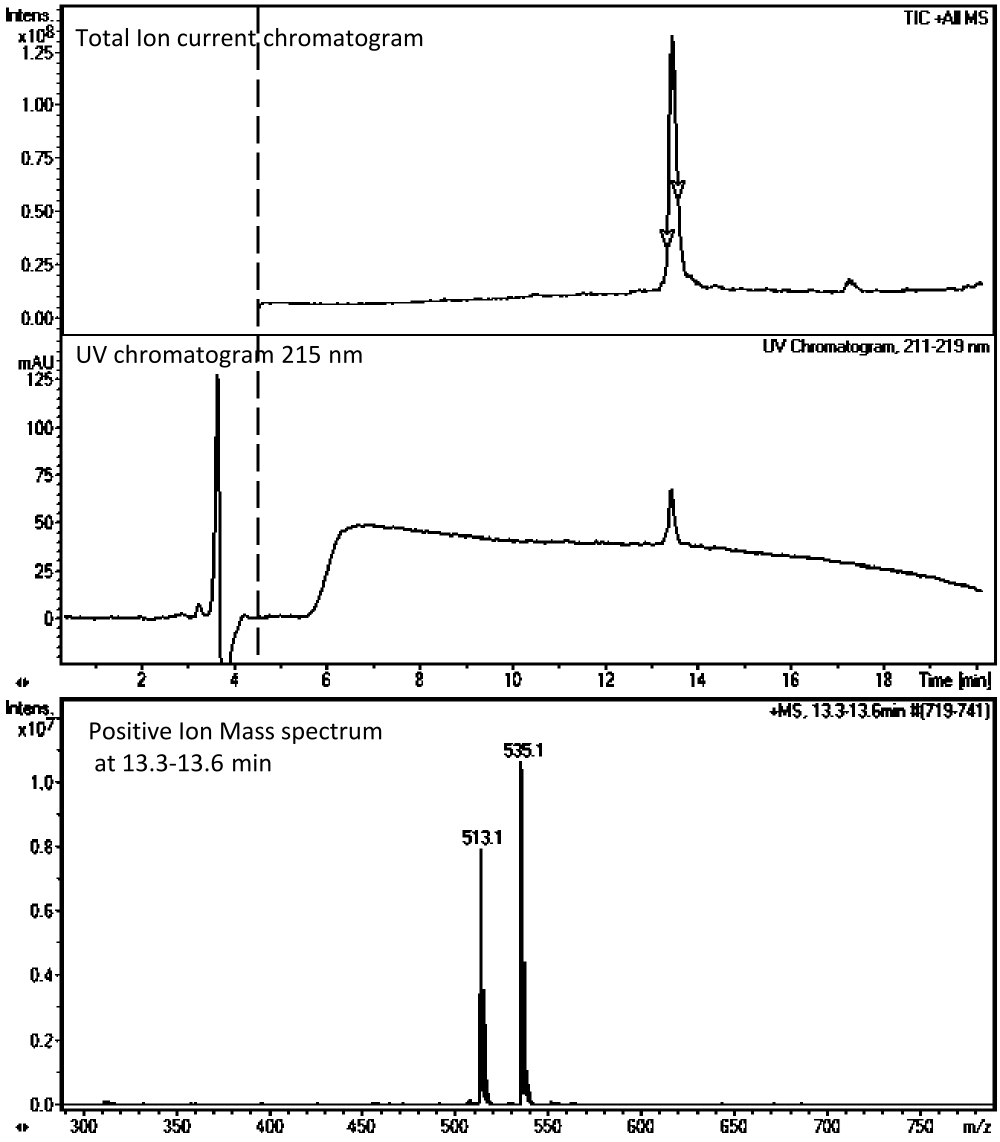

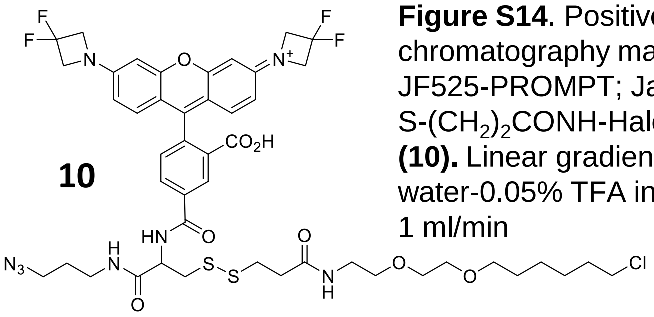

10

Figure S14. Positive ion electrospray liquid chromatography mass spectroscopy analysis of JF525-PROMPT; Janelia Fluor 525-CONH-cys(S-S-$(CH_2)_2$CONH-HaloTag ligand)-CO-NH-$(CH_2)_3$-N_3 **(10).** Linear gradient from 10-100% acetonitrile-water-0.05% TFA in 20mins, then held for 5 mins. 1 ml/min

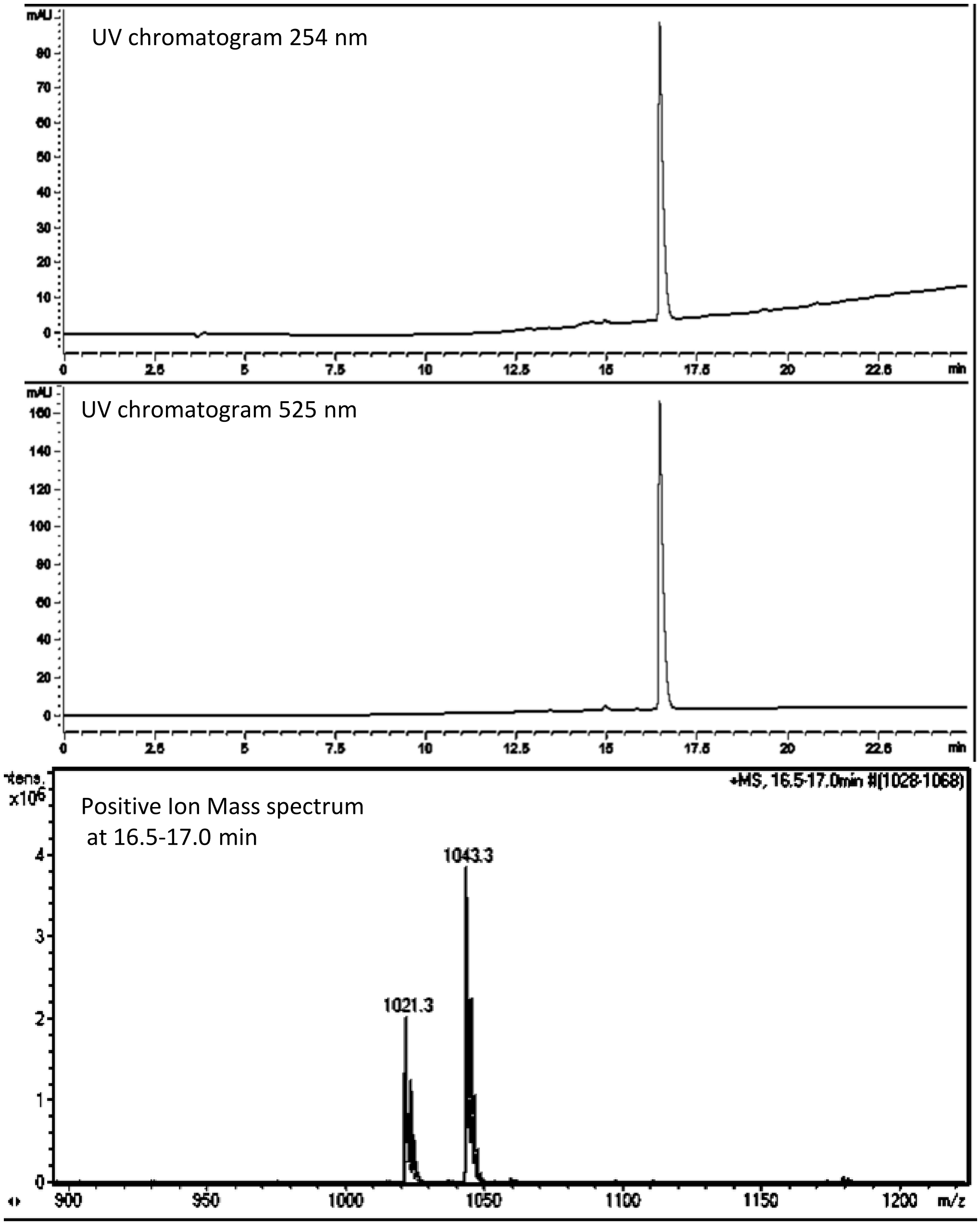

Figure S15. Positive ion electrospray liquid chromatography mass spectroscopy analysis of JF570-PROMPT (**9**) and reaction of reaction of JF570-PROMPT with HaloTag protein. Linear gradient from 20-60% acetonitrile-water-0.05% TFA in 16mins, then to 90% in 4 min, and held for 5 mins. 1 ml/min PLRP-S column

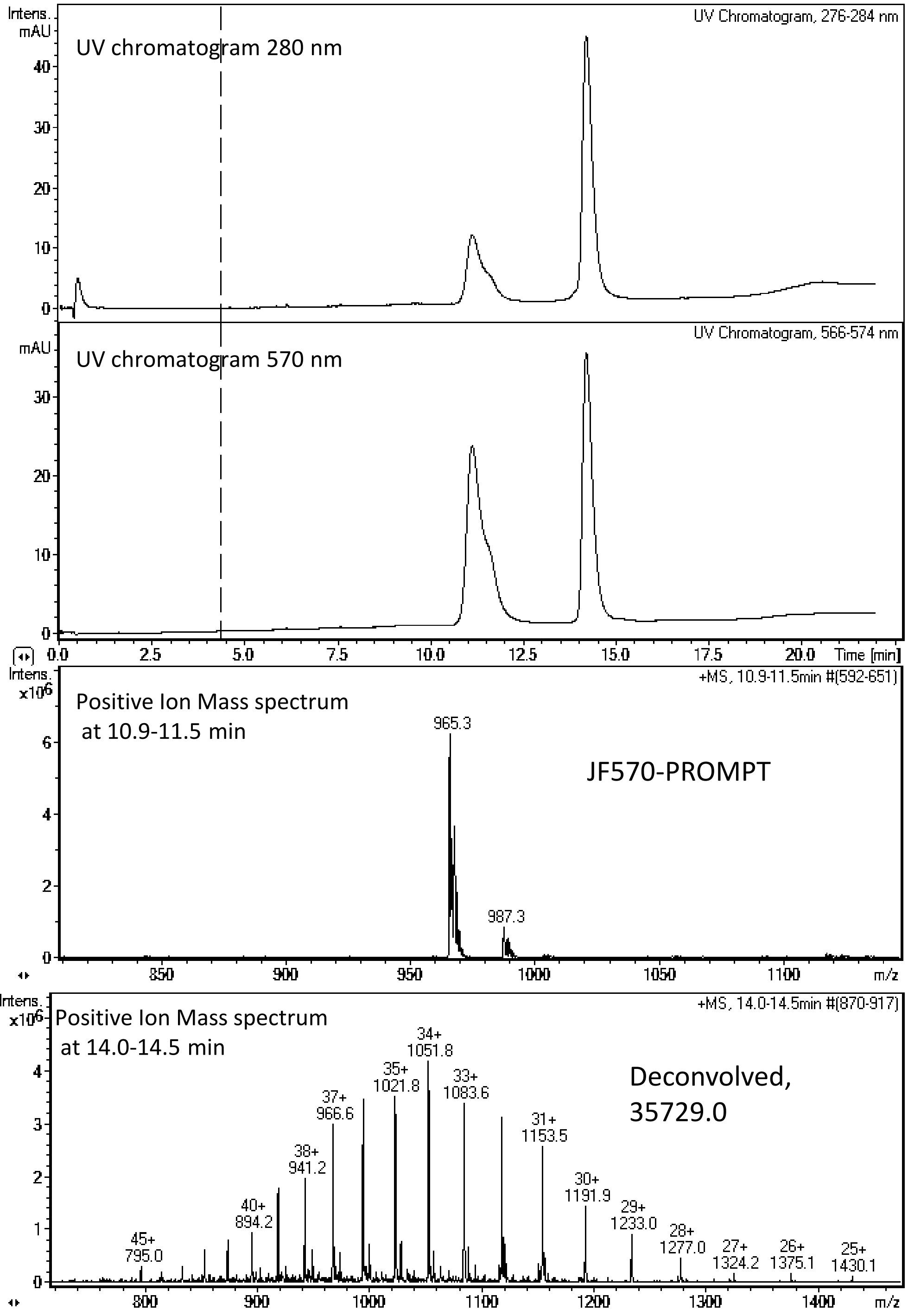

Figure S16. Positive ion electrospray liquid chromatography mass spectroscopy analysis of reaction of JF525-PROMPT with HaloTag protein. Linear gradient from 20-60% acetonitrile-water-0.05% TFA in 16mins, then to 90% in 4 min, and held for 5 mins. 1 ml/min PLRP-S column

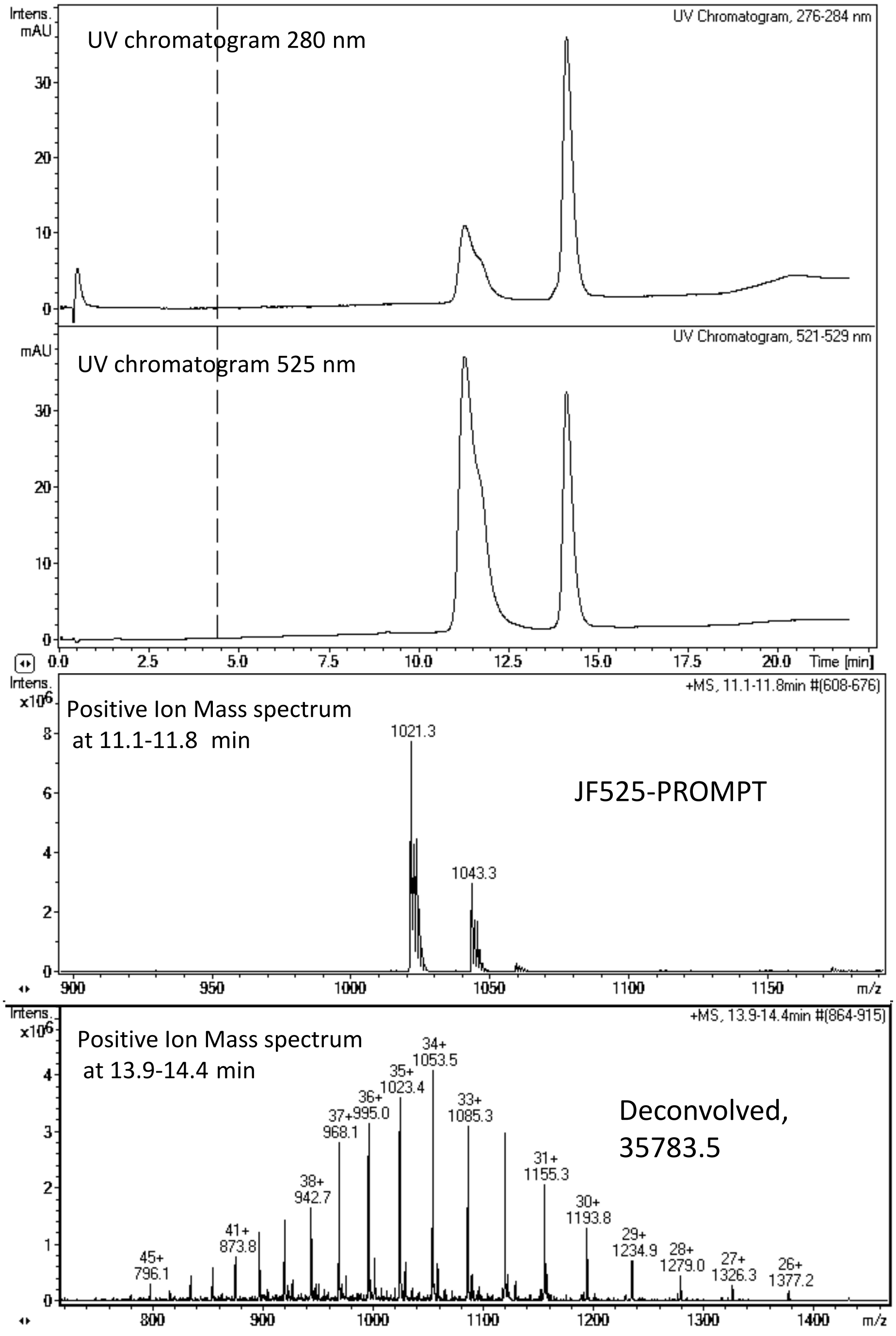

Supplement: Supplementary file 1 — Supplementary Information 1. [file 41598_2023_45413_MOESM1_ESM.pdf]
